# Managing stress and anxiety through qigong exercise in healthy adults: a systematic review and meta-analysis of randomized controlled trials

**DOI:** 10.1186/1472-6882-14-8

**Published:** 2014-01-09

**Authors:** Chong-Wen Wang, Celia HY Chan, Rainbow TH Ho, Jessie SM Chan, Siu-Man Ng, Cecilia LW Chan

**Affiliations:** 1 Centre on Behavioral Health, The University of Hong Kong, 5 Sassoon Road, Pokfulam, Hong Kong, China; 2 Department of Social Work and Social Administration, The University of Hong Kong, Pokfulam Road, Hong Kong, China

**Keywords:** stress, anxiety, qigong, systematic review, meta-analysis

## Abstract

**Background:**

An increasing number of studies have documented the effectiveness of qigong exercise in helping people reduce psychological stress and anxiety, but there is a scarcity of systematic reviews evaluating evidence from randomized controlled trials (RCTs) conducted among healthy subjects.

**Methods:**

Thirteen databases were searched for RCTs from their inception through June 2013. Effects of qigong exercise were pooled across trials. Standardized mean differences (SMDs) were calculated for the pooled effects. Heterogeneity was assessed using the *I*^*2*^ test. The risk of bias was assessed using the Cochrane criteria.

**Results:**

Seven RCTs met the inclusion criteria. Two RCTs suggested that qigong exercise immediately relieved anxiety among healthy adults, compared to lecture attendance and structured movements only. Four RCTs suggested qigong exercise relieved anxiety (pooled SMD = -0.75; 95% CI, -1.11 to -0.40), and three RCTs suggested that qigong exercise reduced stress (pooled SMD = -0.88; 95% CI, -1.22 to -0.55) among healthy subjects following one to three months of qigong practice, compared to wait-list controls.

**Conclusions:**

The available evidence suggests that qigong exercise reduces stress and anxiety in healthy adults. However, given the limited number of RCTs and their methodological flaws, further rigorously designed RCTs are needed.

## Background

Stress is a problem worldwide. People of different ages and backgrounds face stress induced by such factors as workload, studyload, job instability, family responsibilities, conflicts, stressful life events, financial strain, and health problems. A recent survey showed that the majority of Americans were living with moderate or high levels of stress and that about 44% had experienced an increase in stress over the past five years [1]. In highly competitive cities such as Hong Kong, an overwhelming majority of people feel stressed [2].

A certain amount of life stress may be beneficial, but intense or prolonged stress can be harmful and make people feel overwhelmed [3]. The most common reaction to stress is anxiety, and unmanaged stress has a detrimental effect on physical and mental health. It may reduce immune function and result in a range of health problems, such as depression, fatigue, insomnia, headache, stomachache, problematic eating, hypertension, cardiovascular disease, and even cancer [4]. Studies have found that stress contributes to 50% of all illnesses in the US, and that two-thirds of doctor visits were for stress-related illnesses [5].

The best way to manage stress and anxiety is through self-care [3]. In recent years, people have increasingly been using mind-body exercises (such as qigong, tai chi, and yoga) as complementary and alternative therapies to manage psychological stress or anxiety. Qigong exercise is an ancient form of martial arts that was developed in China and has been used in China for thousands of years to improve physical fitness and stamina [6]. The basic components of qigong exercise include concentration, relaxation, meditation, breathing regulation, body posture, and movement [6]. According to the philosophy of traditional Chinese medicine, qigong exercise aims to achieve a harmonious flow of vital energy (*qi*) and regulate the functional activities of the body through regulated breathing, mindful concentration, and gentle movements. With regular practice and rehearsal of the structured movements, as well as concentration on mind and breath, practitioners can experience mood stabilization and improved strength and fitness. Qigong is an easily adaptable form of mind-body exercise that can be practiced any place and any time, without any special equipment. It is widely practiced not just to improve physical health, but also to manage stress and improve psychological well-being.

In recent years, an increasing number of studies have documented the effectiveness of qigong exercise in helping people improve their physical health and reduce perceived stress and anxiety. Existing systematic reviews have examined the clinical evidence of the beneficial effects of qigong exercise on different medical conditions, such as cancer [7], hypertension [8], diabetes [9], chronic heart diseases [10], fibromyalgia [11], and movement disorders [12]. In the most recently published systematic review, our team examined the overall effectiveness of qigong exercise on depressive and anxiety symptoms among patients with chronic illnesses [13]. The results suggested that qigong exercise reduces depressive symptoms but not anxiety symptoms for patients with chronic illnesses. To date, the literature lacks a systematic review of the clinical trial evidence of the effectiveness of qigong exercise on stress and anxiety among healthy people in particular. Thus, the purpose of this systematic review is to summarize and synthesize the clinical evidence available from RCTs on the effectiveness of qigong exercise on stress and anxiety among healthy adults.

## Methods

### The literature search

The following electronic databases were searched: PubMed/MEDLINE; CENTRAL; CINAHL; EMBASE; AMED; Qigong and Energy Medicine Database; China Academic Journals Full-text Database-Medicine/Hygiene Series; China Proceedings of Conference Full-text Database; China Master’s Theses Full-text Database; China Doctoral Dissertations Full-text Database; Taiwan Electronic Theses and Dissertation System; Taiwan Electronic Periodical Services; and Index to Taiwan Periodical Literature System. The search terms included: *qigong, qi-gong, qi gong, chi chung, chi gong, qi chung, qi-training, anxiety, anxious, stress, distress, mood,* and *emotion.* Both traditional and simplified Chinese translations of these terms were used in Chinese databases. We searched the databases from their inception through June 2013 for articles containing these terms in the title, abstract, or keywords. We manually searched the reference lists of all included studies and reviews for other articles.

### Study selection

This study included all RCTs examining the effect of qigong exercise on stress reduction or anxiety relief among healthy adults (defined as those who may have psychological distress but not psychiatric symptoms or chronic illnesses). However, crossover RCTs were excluded because their results are subject to carryover bias. Nonrandomized controlled clinical trials (CCTs) and controlled, retrospective observational studies (ROS) were excluded due to their susceptibility to selection bias. Non-controlled observational studies and case reports were also excluded due to lack of significant evidence. Because the focus of this review was on psychological distress (including stress and anxiety), rather than on psychopathological symptoms, this review excluded studies on patients with mental disorders or patients with elevated depressive and anxiety symptoms secondary to chronic illnesses such as cancer. The results of these studies have already been examined in our prior review [13]. Finally, studies among children and pregnant women were also excluded because our focus was on adults. For each included trial, we extracted data on the effect of qigong on any outcomes examined in the study, although the focus of this review is perceived stress and anxiety.

### Data extraction and risk-of-bias assessment

Data were extracted by one main researcher and then verified by another researcher. Any discrepancies were resolved by discussion. The risk of bias in each of the included trials was assessed using the Cochrane Collaboration’s assessment tool [14]. This tool assesses study quality based on seven criteria: adequate randomization; allocation concealment; blinding of participants, personnel, and outcome assessors; incomplete outcome data reporting; intention-to-treat analysis; selective outcome reporting; and other bias. Since blinding both participants and personnel are generally impossible for studies of qigong exercise, we only assessed if the outcome assessors were blind to treatment allocation. A trial was considered to have used intention-to-treat analysis if all the participants were analyzed with no difference in number between pre- and post-intervention. “Other bias” was assessed mainly based on sample size justification and screening criteria for participants.

### Data synthesis and analysis

Meta-analyses of the results were performed using Review Manager 5.2 ( http://ims.cochrane.org/revman). Effects sizes were calculated for each trial using Hedge’s *g*[15]. Standardized mean differences (SMDs) were calculated for the pooled effects. We interpreted the SMDs using the following rule of thumb: 0.2 represents a small effect, 0.5 a moderate effect, and 0.8 a large effect [16].

A random-effects model was used for data synthesis when an outcome was measured by different measures, and a fixed-effects model was used when an outcome was measured by the same instrument in different studies [14]. A mixture of change-from- baseline scores and final value scores was used for meta-analyses [14]. Where an outcome was assessed by more than one tool in a trial, we only included the main outcome measure (identified as the first outcome reported in the results section or the outcome reported in the abstract) in the meta-analysis. Where both state anxiety and trait anxiety were assessed, we included the measure of state anxiety (anxiety at a moment or about an event) only in the meta-analysis for evaluating the immediate effect of qigong exercise following a single session of practice, and the measure of trait anxiety (anxiety experienced on a day-to-day basis) only in the meta-analysis for evaluating the effect of qigong exercise following a period of qigong practice.

For publications in which means and standard deviations of the outcome measures were not available, we contacted the correspondence authors for the data. The chi-squared statistic and the *I*^2^ statistic were used to assess heterogeneity. Studies with an *I*^*2*^ statistic of >75% were considered to have a high degree of heterogeneity; studies with an *I*^*2*^ statistic of 50-75% were considered to have a moderate degree of heterogeneity; and studies with an *I*^*2*^ statistic of 25-50% were considered to have a low degree of heterogeneity [16]. Sensitivity analyses were conducted by omitting one study in turn and evaluating the influence of a single study on the overall pooled effect. Publication bias was not examined due to the small number of studies (< 10) included in each analysis.

## Results

### Results of the literature search

**Figure 1:**
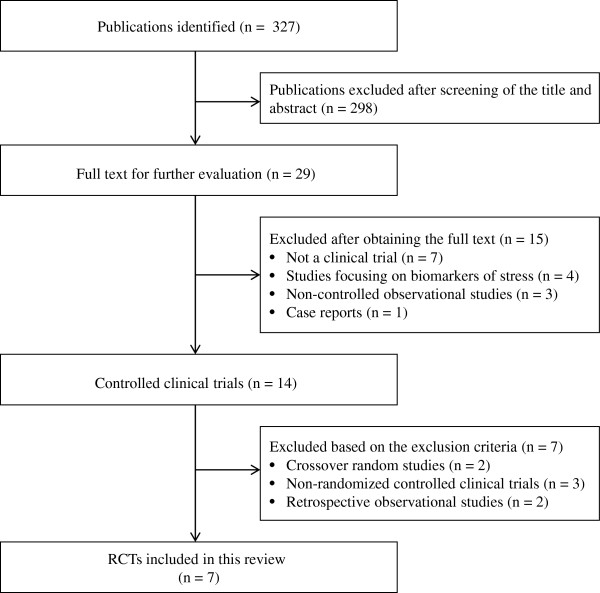
Selection process for included studies.

Our database searches identified 327 potentially relevant articles, of which 298 were excluded after screening the title and abstract. Full reports of 29 studies were acquired, and 22 were excluded based on the inclusion criteria (Figure 1).

### Description of included studies

Seven RCTs [17-23] met the inclusion criteria. They were conducted in Australia [17], South Korea [18,21], Hong Kong [19], the USA [20], Mainland China [22], and Spain [23]. All of them were published in peer-reviewed journals with full texts. Six RCTs were published in English, and one [22] was published in Chinese. Table 1 presents the characteristics of the included studies.

**Table 1:** Summary of seven RCTs investigating the effects of qigong exercise among healthy or distressed individuals

**Studies**	**Design**	**Subjects (age)**	**Sample size (pre/post)**	**Intervention (frequency)**	**Control**	**Duration**	**Relevant outcome measures**	**Results**
Johansson et al., 2008 [17]	RTC	Regular qigong practitioners for 4.8 ± 3.1 years (mean age: 51 years)	QG: 28	Jichu Gong	Lecture attendance	30 min	(1) STAI-state form	(1) *p* < .01
			CG: 31				(2) POMS	(2) *p* < .002 for depression score; *p* < .001 for anger score; *p* < .001 for fatigue score; *p* > .05 for scores of tension, vigor, and confusion.
Lee et al., 2004 [18]	RCT	Healthy male volunteers who were offered 4 weeks of free classes in Qi-training (20–40 years)	QG: 16	ChunDoSunBup Qi training	Structured movements without gathering or moving *qi*.	1 hour	(1) STAI-state form	(1) *p* < .005
			CG: 16				(2) ACTH	(2) *p* < .01
							(3) Cortisol	(3) *p* < .005
							(4) Aldosterone	(4) *p* < .005
Chow et al., 2012 [19]	RCT	Middle-aged adults (21–64 years)	IG: 34/34	Chan Mi Gong (90 min, supervised session once a week for 8 weeks, continued with home practice for 4 weeks)	Wait list	12 wk	(1) DASS-21	(1) DASS-S: *p* = .019; DASS-A: *p* = .034; DASS-D: *p* = .053
			CG: 34/31				(2) ChQOL	(2) *p* = .017
							(3) Salivary cortisol level	(3) *p* < .001
Griffith et al., 2008 [20]	RCT	Hospital staff (mean age: 51 years)	IG: 25/16	Qigong exercise (The Basic Eight qigong) (1 hour, twice a week plus 30 minutes of DVD-directed practice for non-class days)	Wait list	6 wk	(1) PSS	(1) *p* = 0.02
			CG: 25/21				(2) SF-36	(2) *p* = 0.05 for social functioning
							(3) Sleep	(3) NS
Hwang et al., 2013 [21]	RCT	Distressed adults (20–60 years)	IG: 25/19	Brief qigong-based stress reduction program (4 weekly group sessions with a total of 5 hours; 15 min home practice twice daily)	Wait list	4 wk	(1) PSS	(1) *p* < .001
			CG: 25/19				(3) STAI	(2) *p* < .001 for trait anxiety; *p* < .005 for state anxiety
							(3) *Hwa-byung* (anger syndrome) scale	(3) *p* < .05 for personality; *p* < .05 for symptoms
							(4) WHOQOL-BREE	(4) *p* < .05
							(5) Salivary cortisol	(5) NS
Liu et al., 2008 [22]	RCT	College students (Age: n.r.)	IG: 50	Eight-Section Brocade qigong (1.5 hours, 5 times per week)	Wait list	12 wk	SCL-90	*p* < .05 for five of the ten subscales: somatization, obsessive-compulsive, anxiety, depression, and hostility.
			CG: 50					
Manzaneque et al., 2009 [23]	RCT	College students (18–21 years)	IG: 21/16	Eight-Section Brocade (Ba Duan Jin) qigong (30 minutes, 3 group sessions a week plus individual practice on the other days)	Wait list	1 mo	(1) STAI-trait form	(1) *p* < .01
			CG: 18/16				(2) BAI	(2) NS
							(3) BDI	(3) *p* < .01
							(4) PSQI	(4) NS
							(5) TNF-α	(5) NS
							(6) TNF-γ	(6) NS

*CG*, control group; *QG*, qigong group; *RCT*, randomized controlled trial.

*ACTH*, adrenocorticotropic hormone; *ChQOL*, the Chinese quality of life instrument; *DASS*, depression anxiety stress scales; *DASS-A*, DASS-anxiety subscale; *DASS-D*, DASS-depression subscale; *DASS-S*, DASS-stress subscale; *PDS*, psychological distress scale; *POMS*, profile of mood states; *PSS*, perceived stress scale; *SF-36*, 36-item short form health survey; *STAI*: State-trait anxiety inventory; *WHOQOL-BREE*, world health organization quality of life - Abbreviated version.

Participants included regular qigong practitioners [17], healthy volunteers [18], middle-aged adults [19], hospital staff [20], distressed adults [21], and college students [22,23]. The sample sizes ranged from 32 to 100, with a total of 398 participants. This included 199 subjects in the qigong groups and 199 subjects in the control groups. The types of qigong exercise included Jichu Gong [17], ChunDoSunBup Qigong [18], Chan Mi Gong [19], Eight-Section Brocade qigong [20,22,23], and a brief qigong-based stress-reduction program [21]. Durations of group qigong intervention ranged from a single group session of qigong practice to multiple group sessions plus home practice for 12 weeks. All of the studies used a two-armed, parallel-group design. In five prospective RCTs [19-23], qigong was compared to wait-list controls. In two single-session studies [17,18], qigong was compared to active controls.

Regarding outcome measures, perceived stress was assessed in three studies with the Perceived Stress Scale [20,21] and the Depression Anxiety Stress Scale [19]. Six studies assessed anxiety [17-19,21-23]. The anxiety scales included the State-Trait Anxiety Inventory [17,18,21,23], Depression Anxiety Stress Scale [19], and Symptom Checklist-90 [22]. One study reported change-from-baseline scores [19], while others reported scores before and after the interventional program, except for one study [23].

### Effects of qigong exercise on stress and anxiety

Two RCTs [17,18] examined the acute effects of qigong exercise following a single session of qigong practice. One RCT [17] compared qigong exercise to lecture attendance. Another RCT [18] compared qigong exercise to structured movements. Both studies suggested a favorable effect of qigong exercise on state anxiety. Their results were not pooled due to heterogeneity of controls.

**Figure 2:**
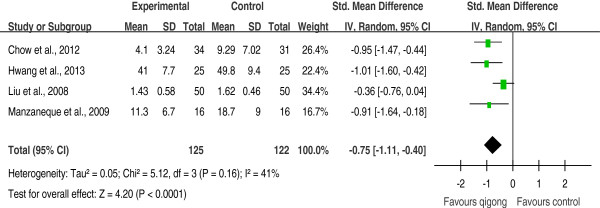
Effects of qigong exercise on anxiety following a period of qigong practice among healthy subjects.

Four RCTs [19,21-23] examined the effect of qigong exercise on anxiety—mainly trait anxiety—following a period of qigong practice. All of them suggested a beneficial effect of qigong exercise on anxiety immediately following the qigong intervention program compared to wait-list controls, and one study [19] suggested that the effect remained 4 weeks later. Their results were pooled, and the pooled SMD was -0.75 [-1.11, -0.40], indicating a significant effect (*p* < 0.001, Figure 2). There was a low degree of heterogeneity (*I*^*2*^ = 41%). Exclusion of any single trial did not significantly alter the pooled effect [SMDs = -0.69 to -0.96, *p* < 0.001].

**Figure 3:**
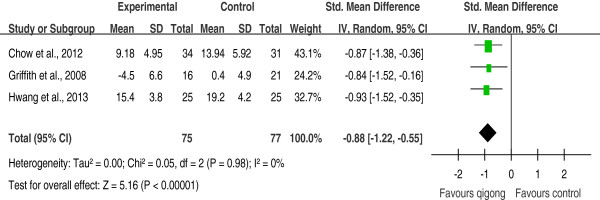
Effects of qigong exercise on perceived stress following a period of qigong practice among healthy subjects.

Three RCTs [19-21] examined the effect of qigong exercise on perceived stress following a period of qigong practice. All three studies suggested a desirable effect of qigong exercise on perceived stress immediately following the qigong intervention program compared to wait-list controls, and one study [19] suggested that the effect lasted 4 weeks. Their results were pooled; the pooled SMD was -0.88 [-1.22, -0.55], indicating a significant effect (*p* < 0.001, Figure 3). There was a high degree of homogeneity (*I*^*2*^ = 0%). Excluding any single trial did not significantly alter the pooled effect [SMDs = -0.86 to -0.90, *p* < 0.001].

### Risk of bias

Table 2 presents the risk-of-bias assessment for each trial. Of the 7 RCTs, randomization method was reported in three trials [18,20,21], but only two used an adequate sequence-generation method for randomization [20,21]. Allocation concealment and blinding of outcome assessors were not used in any of the RCTs. Four studies reported the number of participants that did not complete the intervention program [19-21,23]. Of them, three did not perform intention-to-treat analyses [19,20,23]. One RCT [22] did not report the dropout rate, so we assumed that all participants in the study completed the intervention program. Inclusion and exclusion criteria were adequate in five trials [18-21,23], but only in two trials the participants were screened by stress and anxiety level [19,21]. Sample-size estimation was calculated or justified in only one trial [21].

**Table 2:** Risk-of-bias summary for included randomized controlled trials

**Studies**	**Adequate sequence generation**	**Allocation concealment**	**Blinding of outcome assessors**	**Incomplete outcome data**	**Intention-to-treat analysis**	**Free of selective reporting**	**Free of other bias**
Johansson et al., 2008 [17]	U	U	U	N	Y	N	N
Lee et al., 2004 [18]	N	U	U	N	Y	N	N
Chow et al., 2012 [19]	U	U	U	Y	N	U	Y
Griffith et al., 2008 [20]	Y	U	U	Y	N	Y	U
Hwang et al., 2013 [21]	Y	U	U	Y	Y	Y	Y
Liu et al., 2008 [22]	U	U	U	U	U	N	N
Manzaneque et al., 2009 [23]	U	U	U	Y	N	Y	N

*N*, not applied (high risk of bias); *U*, unclear (uncertain risk of bias); *Y*, yes (low risk of bias).

## Discussion

This systematic review examined and statistically synthesized clinical trial evidence of the effectiveness of qigong exercise at relieving anxiety and reducing stress among healthy individuals. On the basis of the available evidence, our review demonstrated that qigong exercise significantly relieved anxiety and reduced stress among healthy people. Specifically, two RCTs suggested an immediate effect of qigong exercise in reducing anxiety among healthy adults compared to listening to music and structured movements only (pooled SMD = -0.98; 95% CI, -1.42 to -0.54). Four RCTs suggested a favorable effect of qigong exercise on anxiety relief (pooled SMD = -0.75; 95% CI, -1.11 to -0.40) and three RCTs suggested a beneficial effect of qigong exercise on stress reduction (pooled SMD = -0.88; 95% CI, -1.22 to -0.55) among healthy subjects following one to three months of qigong practice, compared to wait-list controls. These results may not be consistent with our findings in patients with depressive and anxiety symptoms secondary to chronic illnesses, which we reported elsewhere [13], but fit with previous systematic reviews of other stress-reduction techniques, such as tai chi [24], yoga [25] and mindfulness-based stress reduction [26] among healthy adults.

However, the results of this review should be interpreted and generalized with caution due to the limited number of the studies and the high risk of bias inherent in the studies. First, qigong exercise was preferentially provided to the intervention groups in these studies as a group therapeutic modality, whereas a matched number of social contact hours with co-participants was not given to the control groups. Thus, a placebo effect might have occurred in participants who enjoyed participating in group activities and being in contact with other people.

Second, of the seven RCTs, only two used an appropriate sequence-generation method for randomization, and none reported adequate concealment of treatment allocation. This might have introduced selection bias. Moreover, blinding of outcome assessors was not used in any of the RCTs, and thus detection bias might have been introduced.

Third, although details of dropouts and withdrawals were described in four RCTs, three studies did not perform intention-to-treat analysis, which might have lead to attrition bias. In addition, only one study justified the sample size; hence, it was unclear if the samples were large enough to avoid Type-II errors for studies with small samples. Finally, most of the studies did not screen participants by stress and anxiety levels, which might have weakened the evidence. These design limitations need to be addressed in future studies.

Given the limited number of RCTs in the field, other controlled studies of the effect of qigong exercise on mood states and stress levels were also examined during the process of our literature review. Results of these studies should be assessed critically. One crossover RCT [27] investigated whether longer qigong sessions have greater acute psychological benefits than shorter sessions. That study suggested that 30 minutes of qigong exercise is sufficient to provide psychological benefits, and it found no additional benefits after 60 minutes, which might provide complementary evidence on the beneficial effect of qigong exercise. Another study [28] with a crossover random design among 42 office workers from the same office suggested no significant effect of qigong exercise on stress reduction. However, the results carry little weight because it might have had carry-over effects and learning effects [29], which could have contributed to the negative result.

We found three CCTs [30-32] and two ROSs [33,34]. Two CCTs [30,31] suggested no effect of qigong on stress reduction, possibly due to the sample size in each of the two trials (n < 20). One CCT [32] suggested a favorable effect of qigong exercise; however, the data were highly susceptible to bias due to the non-random design. Two ROSs [33,34] suggested a "dose–response" effect of qigong exercise in decreasing symptoms of stress, which might provide alternative evidence of the beneficial effects of qigong exercise for stress management. Unfortunately, such data were highly susceptible to bias and provided little scientific evidence.

Assuming that qigong exercise is effective for stress management, possible mechanisms may be of interest. Researchers have speculated that breath regulation and structured body movements during qigong exercise result in long and deep diaphragmatic and rhythmic breathing that could affect the autonomic nervous system (ANS) and the endocrine system, stabilize mood, and restore the homeostatic state by enhancing cardiac output, oxygen consumption, carbon dioxide exhalation, and plasticity of the ANS [24]. Researchers have also proposed three psychobiological pathways (monoamine neurotransmitters in the brain, the hypothalamic-pituitary-adrenal axis, and brain-derived neurotropic factors) to explain qigong exercise’s effects on stress and depression [35]. However, these hypotheses need to be further supported by scientific evidence, given inconsistent findings on stress-related biomarkers reported in three [18,19,21] of the seven RCTs examined in this review.

This review may be subject to several limitations. First is the potential incompleteness of the evidence reviewed, a common concern for any systematic review. The second is that we could not examine the effect of quality and dosage of qigong exercise due to the limited number of RCTs. For the same reason, we could not compare the effect of qigong exercise compared to other intervention modalities, such as psychological education, social support, or aerobic exercise. Finally, we did not synthesize the effect of qigong exercise on other outcomes that may be related to stress, such as sleep quality, quality of life, and stress-related biomarkers due to the limited number of studies. As the number of studies increases, future reviews can address these issues. Despite these limitations, our review is the first to comprehensively and critically assess evidence of the effectiveness of qigong exercise on anxiety relief and stress reduction among healthy subjects, which may provide insight for further studies.

## Conclusions

In conclusion, evidence from a limited number of RCTs suggests that qigong exercise relieves anxiety and reduces stress among healthy individuals. Given the high risk of bias and methodological problems in the RCTs, further rigorously designed RCTs that adhere to accepted standards of trial methodology with large, well-defined samples are warranted before recommending qigong exercise as an intervention option.

## Competing interests

The authors declare that they have no competing interests.

## Authors’ contributions

CLWC was the project leader and initiated the study. CHYC and SMN contributed to the conception of the study. JSMC searched the literature and collected the data. CWW performed the meta-analysis and drafted the manuscript. CLWC, CHYC, RTHH, and SMN contributed comments for revision of the manuscript. All authors read and approved the final manuscript.

## Pre-publication history

The pre-publication history for this paper can be accessed here:

http://www.biomedcentral.com/1472-6882/14/8/prepub
